# Co-Translational Insertion of Aquaporins into Liposome for Functional Analysis via an *E. coli* Based Cell-Free Protein Synthesis System

**DOI:** 10.3390/cells8111325

**Published:** 2019-10-27

**Authors:** Ke Yue, Tran Nam Trung, Yiyong Zhu, Ralf Kaldenhoff, Lei Kai

**Affiliations:** 1 The Key Laboratory of Biotechnology for Medicinal Plants of Jiangsu Province, School of Life Sciences, Jiangsu Normal University, Xuzhou 22116, China; kyue@jsnu.edu.cn; 2 Department of Biology, Applied Plant Sciences, Technische Universität Darmstadt, Schnittspahn Strasse 10, D-64287 Darmstadt, Germany; tran@bio.tu-darmstadt.de (T.N.T.); kaldenhoff@bio.tu-darmstadt.de (R.K.); 3 Jiangsu Provincial Key Lab for Organic Solid Waste Utilization, National Engineering Research Center for Organic-based Fertilizers, Jiangsu Collaborative Innovation Center for Solid Organic Waste Resource Utilization, Nanjing Agricultural University, Nanjing 210095, China; yiyong1973@njau.edu.cn; 4 Department of Cellular and Molecular Biophysics, Max Planck Institute of Biochemistry, D-82152 Martinsried, Germany

**Keywords:** aquaporin, cell-free protein synthesis, co-translational insertion, proteo-liposome

## Abstract

Aquaporins are important and well-studied water channel membrane proteins. However, being membrane proteins, sample preparation for functional analysis is tedious and time-consuming. In this paper, we report a new approach for the co-translational insertion of two aquaporins from *Escherichia coli* and *Nicotiana tabacum* using the CFPS system. This was done in the presence of liposomes with a modified procedure to form homogenous proteo-liposomes suitable for functional analysis of water permeability using stopped-flow spectrophotometry. Two model aquaporins, AqpZ and *Nt*PIP2;1, were successfully incorporated into the liposome in their active forms. Shifted green fluorescent protein was fused to the C-terminal part of AqpZ to monitor its insertion and status in the lipid environment. This new fast approach offers a fast and straightforward method for the functional analysis of aquaporins in both prokaryotic and eukaryotic organisms.

## 1. Introduction

Although the function of aquaporins (AQPs) as water channel proteins has been well studied for decades, it is a challenge to produce sufficient amounts of high-quality protein samples for applications such as creating bio-membranes for water filtration. Recent advances in material science have resulted in the creation of a variety of block co-polymers that are useful as artificial membranes for the integration of functional AQPs [1,2]. However, the production of large amounts of functional aquaporin, as well as their integration into the designed bilayers, can pose significant bottlenecks for such applications. Conventional cell-based expression systems are often unsuitable for producing membrane proteins due to their toxicity to host cells. The Cell-Free Protein Synthesis (CFPS) system has, therefore, emerged as an alternative option for the production of such difficult proteins [3,4,5], with particular focus on membrane proteins [6,7].

Previous studies have shown that high amounts of functional AQPs from both prokaryotic [8] and eukaryotic organisms [9,10,11,12] are produced in CFPS systems. While the initial production of AQPs may also be efficient in cell-based systems [9,13], their subsequent integration into defined lipid bilayers can still be problematic and time-consuming. The open nature of CFPS systems allows the design of a variety of defined lipid environments directly in the expression reaction. The CFPS of membrane proteins in the presence of supplied lipids (l-CFPS mode) can promote the co-translational insertion of AQPs into the provided membranes [14].

In this paper, the AQPs, AqpZ from *E. coli* and *Nt*PIP2;1 from *Nicotiana tabacum*, were used as model proteins to investigate the process of their co-translational integration into liposomes. Improved homogeneity of the resulting aquaporin proteoliposomes was achieved via an interim detergent solubilization step. A shifted green fluorescence protein (sGFP) fused to the C-terminal of AqpZ was used as a monitor to follow the process of integration via confocal fluorescence microscopy. The function of water permeability was determined via stopped-flow spectrophotometry. The obtained results indicate a comparable functional incorporation of both aquaporins to conventional reconstitution methods. We demonstrate the functional insertion of both selected model AQPs from different organisms and present a simplified and streamlined protocol with improved efficiency.

## 2. Materials and Methods

### 2.1. Plasmid and DNA Manipulation

cDNA sequence of *Nt*PIP2;1 was amplified from yeast expression vectors created previously [15] using the following pair of primers: 5′- AAAAGAATTCATGTCAAAGGACGTGATTG-3′, 5′- AAAA CTCGAGGTTGGTTGGGTTACTGC-3′. Restriction sites are shown in bold. Digested PCR products were ligated with a linearized pET21a vector, resulting in a poly(His)6 tag at the C-terminal of *Nt*PIP2;1. AqpZ-sGFP was constructed as reported before [7] and expressed from the vector pET28(a+) containing a C-terminal TEV protease recognition sequence (ENLYFQ/G), followed by a C-terminally His_8_-tagged GFP. DNA templates used for CFPS were transformed into *E. coli* strain DH5a and isolated by standard plasmid purification kits (Macherey-Nagel, Düren, Germany).

### 2.2. CFPS

The CFPS system was constructed using a self-prepared *E. coli* A19 extract. The preparation of S30 extracts, T7 polymerase, and basic compounds was prepared as reported before [5,16,17]. A continuous exchange cell-free setup was used, with a reaction mixture (RM) volume of 55 μL. A ratio of 1:15 of RM and feeding mixture (FM) was used. RM and FM were transferred into homemade mini-reactors with membranes of regenerated cellulose and a molecular cut-off of 12-14 kDa [6]. The mini-reactors were then incubated overnight at 30 °C with gentle shaking. For CFPS with Brij^®^ S20, a final concentration of 1% (*w*/*v*) was introduced into both RM and FM, substituting the extra volume of water. For CFPS in the presence of lipids (l-CFPS), preformed liposomes were added into the RM with corresponding final concentrations, substituting the volume of water.

### 2.3. Liposome Preparation

l-α-Phosphatidylcholine from soybean and detergent used in this study were purchased from Sigma-Aldrich (Taufkirchen, Germany). Lipid mixtures solubilized in chloroform were first transferred to a round-bottom flask. A thin lipid film was formed by rotary evaporation under a controlled vacuum. The flask was then placed in a vacuum chamber overnight. The lipids were rehydrated in 1 mL of assay buffer A (50 mM HEPES-KOH, pH 7.5, 50 mM NaCl) to a final concentration of 40 mg/mL. The mixture was vortexed for 15 min to form multilamellar vesicles. The multilamellar vesicle solution was passed at least 21 times through an Avanti Polar Lipids (Alabaster, AL, USA) mini extruder holding a 200-nm Whatman polycarbonate membrane filter (Florham Park, NJ, USA) sandwiched with two filter supports on each side. The resulting unilamellar liposome solution was used for expression or water permeability experiments.

### 2.4. Fluorescence Quantification

AqpZ-sGFP was quantified by fluorescence measurement with excitation wavelength of 484 nm and emission wavelength of 510 nm [17]. Further method parameters were defined in TECAN Magellan 5.03 software: Gain (Manual): 25; number of reads: 10; integration time: 40 ms; lag time: 0 ms; mirror selection: automatic; multiple reads per well (Circle): 262; incubation time: 20 s; settle time: 20 s.

### 2.5. Sample Preparation for Water Permeability Measurements

In vitro reconstitution of purified AqpZ-sGFP into liposomes composed of l-α-Phosphatidylcholine from soybean was performed as previously described [9,12], with modifications. In brief, a reconstitution mixture was prepared in a microtube at room temperature by sequentially adding assay buffer A, 10% (*v*/*w*) Triton X-100 (final concentration 4 mM), 20 mg/mL preformed liposomes (final concentration 4 mg/mL), and 200 µg/mL purified AqpZ-sGFP. The reconstitution mixture was incubated at room temperature with gentle shaking for 30 min. The detergent was removed by SM-2 beads (Bio-Rad, München, Germany) according to the manual. Finally, the liquid reconstitution mixture was sent to ultracentrifugation at 500,000× *g* for 45 min. Then, the pellet was washed again with reconstitution buffer. After the wash step, the proteo-liposome solution was ultra-centrifugated again and finally re-suspended in 1.6 mL reconstitution buffer. Samples from the CFPS reaction were treated following the steps described below:

After overnight expression, the mixture was pelleted down via centrifugation at 18,000× *g* for 20 min. The pellet fraction containing precipitated protein, and protein inserted into liposomes and fused liposomes were collected. The supernatant, which did not contain any expressed protein, and a small amount of lipids was discarded. Pellet fractions were re-suspended in assay buffer A. In order to separate the precipitated proteins from proteins that were inserted and associated with lipids, detergents (TritonX100 with final concentration of 0.42% (*w*/*v*) or 1% (*w*/*v*) Octyl β-d-glucopyranoside (β-OG)) per 4 mg/mL of lipids were used to solubilize the pellet of lipids; the precipitated protein remained insoluble. After detergent re-solubilization, the sample was centrifuged again at 18,000× *g* for 10 min. The supernatant was collected and subjected to either dialysis or bio-beads treatment for detergent removal. For dialysis, a stepwise process was applied with 0.5%, 0.25%, 0.125%, and 0% β-OG final concentration in assay buffer A. Each dialysis was for around 6 h at 4 °C, with a final additional dialysis step for 12 h to completely remove the detergent. For the bio-beads treatment, 40 mg bio-bead per 1 mg/mL lipids reaction was applied, and the bio-beads were pre-incubated with 4 mg/mL liposome in assay buffer A incubation overnight. The proteo-liposome was reformed after removal of the detergent. All the samples with different treatments were extruded through a 200-nm membrane filter before application of the stopped-flow measurements.

### 2.6. Preparation of Giant Unilamellar Vesicles (GUVs) from Large Unilamellar Vesicles (LUVs)

The formation of giant liposomes for confocal fluorescence microscopy was performed by following a modified protocol based on a description by Tsumoto et al. [18]. In brief, 25 µL lipid sample (concentration: 4 mg/mL) was mixed with 25 µL glucose solution (concentration 9.3 mg/mL in Assay buffer A) and 1 µL NaN_3_ 10%. The lipid-to-glucose molar ratio was 1:10. The mixture was treated with three cycles of freeze/thaw: 1 min freezing in liquid N_2_ followed by 5 min thawing at 42 °C (water bath), with vortexing between cycles. The sample was subsequently put on a siliconized glass slide and dried under the hood for several hours. In the next step, dried lipid film was covered with 25 µL MilliQ water and made to sit overnight in a humid closed petri dish (by putting pieces of wet paper tissue under the glass slide). The samples were collected and transferred into a new tube the following day.

### 2.7. Water Permeability Measurements

The setup of stopped-flow spectrophotometry was done as per a previous report [12]. In brief, liposomes or proteo-liposomes were measured at 436 nm in a stopped-flow spectrophotometer (SFM 300, Bio-Logic SAS, Claix, France). Sample suspensions were quickly mixed with equal volumes of hyperosmotic solution (assay buffer A with 400 mM sucrose). Data obtained from the spectrophotometer was fitted into an exponential rise equation; the initial shrinkage rate (*k*) was determined by the fitted curve of 6–10 measurements. The water permeability factor (*P*_f_) of the vesicle samples was calculated using the equation described previously [9,12,19]:

(1) $$P_{f}=\frac{k}{(S/V_{0})\times V_{w}\times {∆}_{osm}}$$

where *S*/*V_0_* is the vesicle’s initial surface-to-volume ratio, *V_w_* is the partial molar volume of water (18 cm^3^/mol), and ${∆}_{osm}$ is the osmotic driving force. The *S*/*V*_0_ was calculated by determining the diameters of the proteo-liposomes using dynamic light scattering (ZetaPlus particle sizing software 2.27). The ${∆}_{osm}$ was 200 mM in this case.

### 2.8. Confocal Fluorescence Microscopy

Nile red (Sigma-Aldrich, Germany) was used to stain the lipid molecules. In brief, 5 µL of the sample, 4 µL of the DABCO solution in water (20 mg/mL), and 1 µL of the Nile Red solution in DMSO (1%) were mixed together and incubated for 30 min. The specimens were covered by High Precision Microscope Cover Glasses (20 × 22 mm, thickness 170 +/− 5 µm) and sealed on four sides with nail polish.

Confocal images were acquired using a TCS SP5 II (Leica) confocal laser scanning microscope equipped with a 100/1.4NA oil immersion objective. sGFP, Nile red was excited at 488, 561 nm laser. All images were recorded in room temperature using Image Processing Leica Confocal and Fiji [20].

## 3. Results

### 3.1. Co-Translational Incorporation of AqpZ-sGFP into Preformed Liposomes

**Figure 1:**
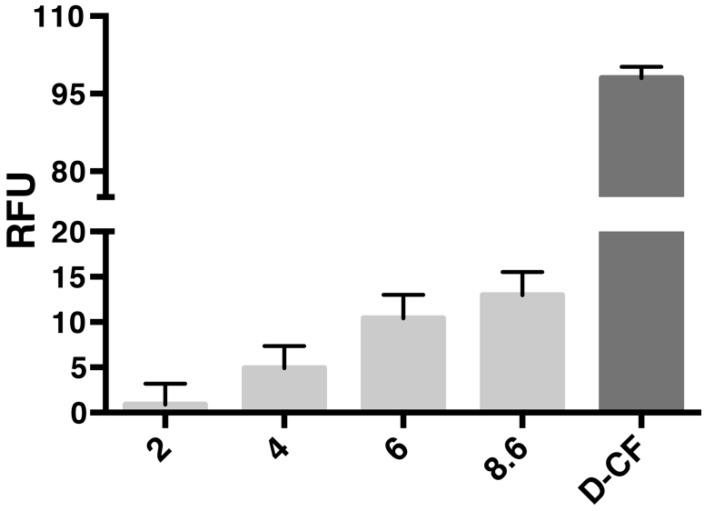
Screening of liposome concentration for CF expression of AqpZ-sGFP. The final concentrations of lipids were indicated with numbers below the bar chart. The reaction mixture with 8.6 mg/mL final lipid concentration without the AqpZ-sGFP plasmid template was used as the negative control. D-CF sample was used as positive control. RFU, relative fluorescent unit.

l-CFPS reaction conditions were used according to the previous description [9,12], including the Mg^2+^, K^+^ and plasmid concentrations. The fusion construct of AqpZ-sGFP was expressed in the presence of liposomes (l-α-Phosphatidylcholine from soybean) with final concentrations up to 8.6 mg lipid per ml reaction mix (RM). After overnight incubation at 30 °C, the fluorescence of the RM was measured in order to determine the formed AqpZ-sGFP proteo-liposomes. Empty reactions without a plasmid template at a lipid concentration of 8.6 mg/mL were used as the negative control. CFPS of AqpZ-sGFP in the presence of detergent (d-CFPS) with final concentrations of 1% Brij^®^ S20 was used as positive control. Within the analyzed range, the fluorescence signal was in a linear correlation with the lipid concentration (Figure 1.). Therefore, the highest possible lipid concentration was used in further experiments.

**Figure 2:**
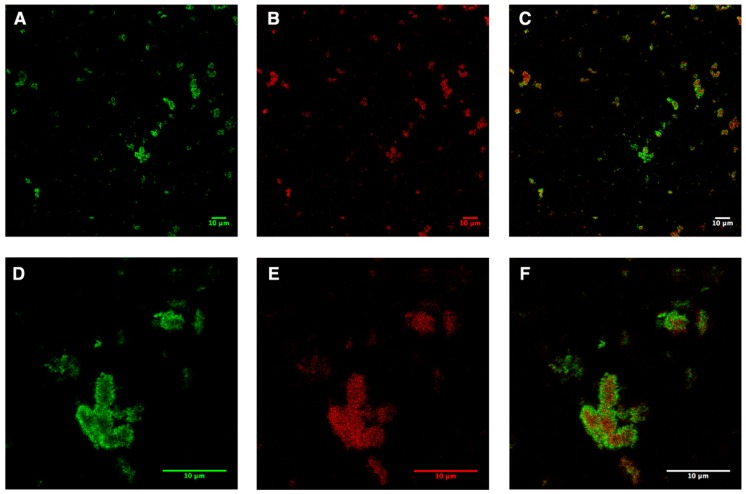
Confocal microscopy analysis of AqpZ-sGFP pellet fractions produced from l-CFPS directly after expression. (**A**,**D**), signals of sGFP in the pellet fractions shown in green; (**B**,**E**), signals of lipids in the pellet fractions in red (stained via Nile red); (**C**,**F**), merged images of the green and red channels. Scale bar indicates 10 μm.

Due to the fusion and instability of the preformed liposomes, a pellet mixture was formed, which consisted of empty liposomes, AqpZ-sGFP proteo-liposomes, precipitated protein; AqpZ-sGFP was only associated (not integrated) with the liposomes after overnight incubation. In order to monitor the distribution of AqpZ-sGFP, the liposomes were stained with Nile red, as described in the Methods section. In brief, the green channel showed the signal from sGFP, while the red channel showed the signal from Nile Red. The overlay of both channels indicates the distribution of AqpZ-sGFP in the liposomes. The liposome pellet obtained directly after the expression was inhomogeneous with randomly incorporated AqpZ-sGFP (Figure 2). Large clusters of fused liposomes up to 10 μm in diameter were visible. Although the sGFP signal was distributed all over the lipid signal in red, a clear cluster of AqpZ-sGFP was observed at the edge of the lipid clots.

### 3.2. Improving Homogeneity of l-CFPS Produced AqpZ-sGFP Proteo-Liposomes for Functional Analysis of Water Permeability

**Figure 3:**
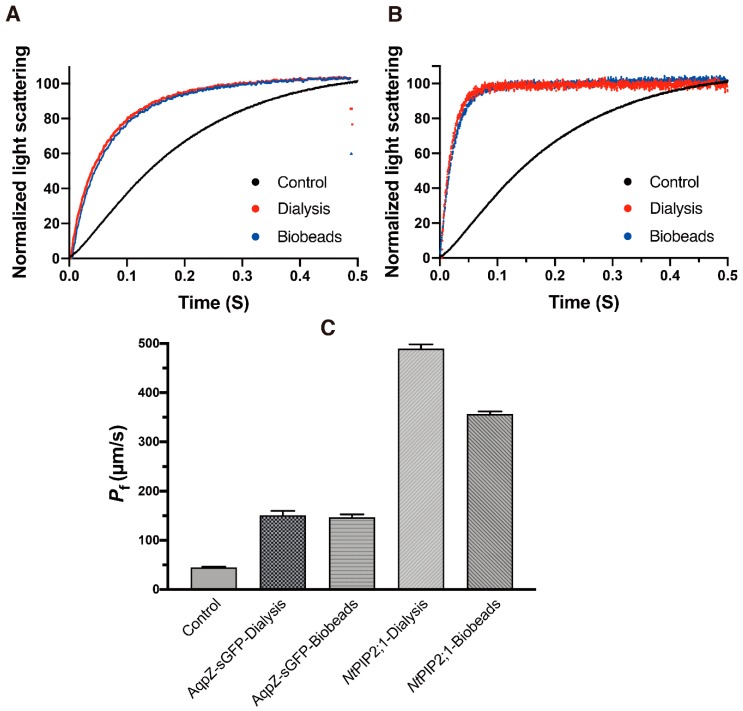
Water permeability of AqpZ-sGFP and *Nt*PIP2;1 proteo-liposome reformed via a detergent re-solubilization of the L-CFPS produced sample. (**A**,**B**), normalized and averaged light scattering measurements of AqpZ-sGFP and *Nt*PIP2;1 proteo-liposome under osmotic shock via a stopped-flow spectrophotometry (*n* = 8–10). Red trace, proteo-liposome formed via the step dialysis method; blue trace, proteo-liposome formed via bio-beads; black trace, control empty liposomes. (**C**), bar chart of the calculated water permeability *P*_f_ (mean ± SD) of corresponding samples.

In order to improve the homogeneity of the l-CFPS produced sample, a detergent solubilization step was introduced. Two commonly used detergents, Triton™X-100 and Octyl-β-d-glucopyranoside (β-OG), were selected to solubilize the pellet fraction. The final concentration of 0.42% Triton™X-100 and 1% β-OG for 4 mg/mL l-α-phosphatidylcholine (PC) were used (See Supplemental Materials for the titration experiment [21].) Then, two methods were applied to remove the detergents and reform the proteo-liposomes. For removal of Triton™X-100, bio-beads were used. For removal of β-OG, stepwise dialysis was used (see Methods for more details). After the detergent was removed, the sample was extruded through a polycarbonate membrane filter with a pore size of 200 nm. The size of the resulting proteo-liposome was determined via dynamic light scattering (indicated in Table 1). In parallel, water permeability of the AqpZ-sGFP proteo-liposome from the two detergent removal methods was determined via stopped-flow spectrophotometry. The functional analysis of water permeability was shown in Figure 3A. The AqpZ-sGFP proteo-liposomes showed increased water permeability (*P*_f_) of 150.60 ± 9.33 μm/s and 146.61 ± 6.03 μm/s, increasing to more than three folds if compared to the *P*_f_ value of 44.88 ± 1.33 μm/s for empty liposomes (control empty liposome was prepared following the same procedure as those of AqpZ and *Nt*PIP2;1, only plasmid template was replaced with water) (see Table 1). Furthermore, we employed a water-specific channel *Nt*PIP2;1 from *Nicotiana tabacum* to validate this method. Normalized light scattering measurements were shown in Figure 3B; a similar result was obtained wherein the reformed proteo-liposome also showed an increased water permeability with corresponding detergent removal methods. The water permeability was even higher with *Nt*PIP2;1 proteo-liposomes—more than 10 times as much as that of control empty liposomes (See Figure 3C and Table 1).

### 3.3. Visualization of Aquaporin Insertion

**Figure 4:**
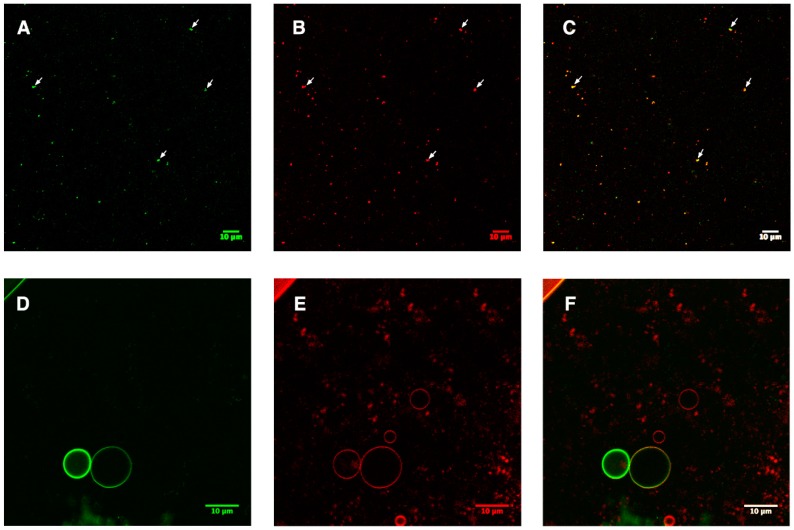
Monitoring the insertion of AqpZ-sGFP after reforming of the proteo-liposome. sGFP and lipids (stained via Nile red) are shown in green and red; a merged image is shown in C and F. (**A**–**C**), representative sample of the AqpZ-sGFP proteo-liposome formed after removal of detergents either via dialysis or bio-beads. The white arrows indicate the position of co-localized signals. (**D**–**F**), GUVs formed via fusion of AqpZ-sGFP proteo-liposome.

Via fusion with the sGFP label, the insertion of aquaporin could be monitored via confocal microscopy. As shown in Figure 4A–C, an overlap of the green and red channels indicated the successful incorporation of AqpZ-sGFP into liposomes (representative overlapping signals are pointed out with white arrows). A fraction of those LUVs was processed to form GUVs using the hydration method described in the Methods section. As shown in Figure 4D–F, overlap of the green sGFP signal with the red lipid signal of GUVs further confirmed the successful insertion of AqpZ-sGFP. This offers direct evidence of the successful formation of Aqp-sGFP proteo-liposomes by introducing a detergent re-solubilization step and reforming LUVs suitable for functional analysis.

## 4. Discussion

In previous studies, the heterogeneous expression of recombinant AQPs using different expression systems was found to be successful, i.e., *E. coli* [22], yeast [23], *Xenopus oocyte* [24], and insect cells [25]. Especially with CFPS systems, a large amount of functional AQPs can be produced in a short period of time—from 4 h to overnight [8,9,10,26]. Still, the integration of AQPs into the lipid bilayer to form a proteo-liposome was a bottleneck for fast and robotic functional analysis. In vitro reconstitution of membrane proteins into liposomes was well established, i.e., organic solvent-mediated and detergent-mediated reconstitution [27]. However, due to the risk of denaturation on exposure to organic solvents as well as on forming multilamellar structures, solvent-mediated reconstitution was used only in limited cases [27]. Instead, the detergent-mediated reconstitution method had better compatibility with membrane proteins and was widely used for in vitro reconstitution. During the reconstitution process, the selection and removal of detergents were crucial steps. However, no universal protocols exist. Each specific target had to undergo the same tedious optimization every time [10,28]. In this work, by embracing the advantage of the CFPS system being an open expression system, liposomes were directly introduced into the reaction mixture. Newly expressed AQPs can obtain direct contact with the lipid environment and be incorporated into liposome co-transnationally. Using this approach, the routine step of purification, therefore, can be avoided.

By applying liposome into the CFPS reaction mixture, pellet fractions were formed after overnight incubation. Previous research using a batch CFPS system was able to obtain AqpZ proteo-liposome with increased water permeability using slow centrifugation, followed by a dialysis step [8]. However, in our continue exchange CFPS set-up, it was not possible to apply this separation method via low speed centrifugation because most of the vesicle precipitated in the pellet fraction [14]. Another study using the same system showed that synthetic liposomes alone tend to fuse and precipitate under cell-free expression conditions [14]. This might be due to the existence of high concentrations of Mg^2+^ ions and PEGs in the micro-environment of the CFPS system, both of which were routinely used for the fusion of liposomes [29,30]. As indicated in Figure 2, AqpZ-sGFP was successfully expressed and stayed in the vicinity of the lipids. A similar sample status was observed with l-CFPS-produced GPCRs in previous reports [31]. As shown in Figure 2, the direct view of the pellet fractions indicated that the sample collected from pellet fractions of the CFPS reaction mixture consisted of inhomogeneous proteins and lipids. This explained the failure of isolation of proteo-liposomes via density ultracentrifugation, as well as extrusion of the pellet fractions through a polycarbonate membrane filter to reform homogenous proteo-liposomes (data not shown).

In order to reform homogenous proteo-liposomes, a step of re-solubilization via detergent was then introduced. According to previous research, membrane proteins expressed in the CFPS system as precipitate can be selectively resolubilized with certain detergents [32]. In contrast, the lipids together with incorporated protein can be completely solubilized with all kinds of detergents [28,33]. The process of detergent re-solubilization can be used as a separation method to isolate the inserted membrane protein from the precipitated proteins. After separation, the classical detergent removal method can be used to reform homogenous proteo-liposomes, which could be suitable for functional analysis using stopped-flow spectrophotometry.

Quite a number of detergents like Triton™X-100, DDM, DM, and β-OG, among others, were used for membrane protein reconstitution [28]. Previous reports showed that Triton™X-100 had better performance in membrane reconstitution via bio-beads, while DDM tend to disrupt the structure of the liposome at high concentrations [28,34,35]. In addition, based on previous reports on the in vitro reconstitution of aquaporins using Triton™X-100 [9], Triton™X-100 was selected instead of DDM in this study. β-OG was selected as the second detergent because it is often used as the detergent for in vitro reconstitution, followed by dialysis as the detergent removal method [36]. From the representative fluorescent image of AqpZ-sGFP in Figure 4, both methods for detergent removal were successful in reforming homogenous proteo-liposomes. Stepwise dialysis to remove β-OG showed a slightly higher water permeability, similar to that with bio-beads. Similar findings (that stepwise dialysis could yield high integration) were also reported from Kumar et al. [37]. Results from the water permeability assay show that the *Nt*PIP2;1 proteo-liposome had a much higher value, when compared to that of the AqpZ-sGFP proteo-liposome. This might result from the influence of the fused sGFP, disrupting the formation of the stable tetramer [22].

With a streamlined protocol to express and integrate aquaporins into proteo-liposomes, functional analysis of water permeability could become more robust, which certainly will boost downstream applications such as manufacturing of biomimetic water filtration membranes [38,39], as well as other pharmaceutical-related studies [40]. However, the high cost of the CFPS system needs to be considered when a large amount of functional proteins was required; this could pose a challenge for the application of CPFS in other fields.

## 5. Conclusions

The present modified approach to create proteo-liposomes with functional aquaporins using the l-CFPS mode demonstrated a fast method for functional analysis of aquaporins. The key stage of detergent re-solubilization of the l-CFPS pellet fraction provides a single-step separation of precipitated and incorporated aquaporin. The results from both confocal microscopy image and stopped-flow spectrophotometry show the successful and functional incorporations of two model aquaporins into homogenous LUVs. Flexibility in the selection of detergents, lipids, and reforming proteo-liposomes could enable further study of other membrane proteins in various lipid environments.

## Figures and Tables

**Table 1:** Water permeability from different samples.

Sample	Diameter (nm)	k	Pf (μm/s)	Normalization (%)
Control	180	5.38 ± 0.16	44.88 ± 1.33	100
AqpZ-sGFP-Dialysis	210	15.49 ± 0.96	150.60 ± 9.33	335.6
AqpZ-sGFP-Biobeads	210	15.08 ± 0.62	146.61 ± 6.03	326.7
NtPIP2;1-Dialysis	195	54.23 ± 0.99	489.58 ± 8.94	1090.9
NtPIP2;1-Biobeads	195	39.49 ± 0.59	356.51 ± 5.33	794.4

## Supplementary Materials

The following are available online at https://www.mdpi.com/2073-4409/8/11/1325/s1, Figure S1: Titration of liposome with detergents [40].
