# Supplementary material for: Co-Translational Insertion of Aquaporins into Liposome for Functional Analysis via an E. coli Based Cell-Free Protein Synthesis System
Source: Cells. 2019 Oct 27;8(11):1325. doi: 10.3390/cells8111325 (PMC6912355; doi:10.3390/cells8111325)
Supplement: Supplementary file 1 [file cells-08-01325-s001.pdf]

Supplementary information:

Co-Translational Insertion of Aquaporins into Liposome for Functional Analysis via an *E. coli* Based Cell-Free Protein Synthesis System

Ke Yue [1], Tran Nam Trung [2], Yiyong Zhu [3], Ralf Kaldenhoff [2] and Lei Kai [1,2,4,*]

[1] The Key Laboratory of Biotechnology for Medicinal Plants of Jiangsu Province, School of Life Sciences, Jiangsu Normal University, Xuzhou 22116, China; kyue@jsnu.edu.cn

[2] Department of Biology, Applied Plant Sciences, Technische Universität Darmstadt, Schnittspahn Strasse 10, D-64287 Darmstadt, Germany; tran@bio.tu-darmstadt.de (T.N.T.); kaldenhoff@bio.tu-darmstadt.de (R.K.)

[3] Jiangsu Provincial Key Lab for Organic Solid Waste Utilization, National Engineering Research Center for Organic-based Fertilizers, Jiangsu Collaborative Innovation Center for Solid Organic Waste Resource Utilization, Nanjing Agricultural University, Nanjing 210095, China; yiyong1973@njau.edu.cn

[4] Department of Cellular and Molecular Biophysics, Max Planck Institute of Biochemistry, D-82152 Martinsried, Germany

* Correspondence: lkai@jsnu.edu.cn or kai@biochem.mpg.de; Tel.: +49-(0)89-8578-2319

Solubilization of liposome via detergents

Titration experiments were performed for liposomes prepared from L-α-Phosphatidylcholine using β-OG (20%) and TritonX-100 (5%). Optical density at 436 nm was used for lipid-detergent interaction determination according to previously reported [1].

Titration of OG and TX-100 was performed by adding β-OG (20%) or TX-100 (5%) solutions to 200 μL suspension of liposome (4 mg/mL in Assay buffer A) in increments of 2 μL. OD_{436nm} was measured immediately after each step. Titration curves were generated by plotting detergent-to-lipid ratios against corresponding OD_{436nm}. Detergent working concentration was chosen as the point at which liposomes were completely solubilized and OD_{436nm} reduced to almost zero. Titration curves were shown in Figure S1.

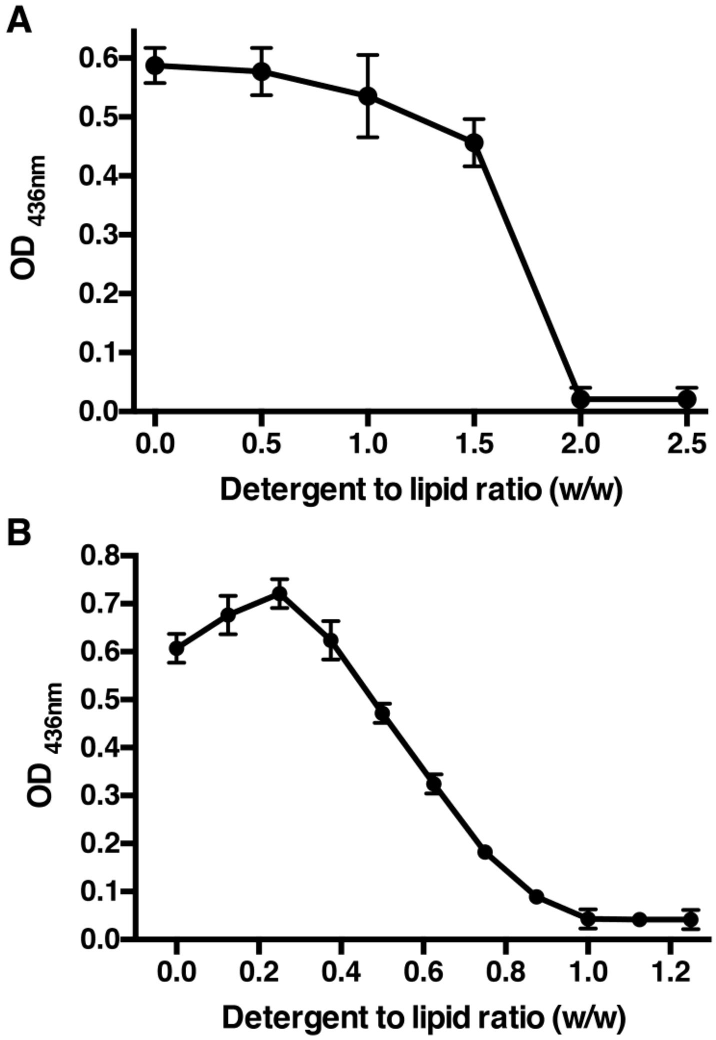

Figure S1. Titration of liposome with detergents. A, titration with β-OG B, titration with TritonX-100.

Reference:

1. Matsuzaki, K.; Murase, O.; Sugishita, K.; Yoneyama, S.; Akada, K.; Ueha, M.; Nakamura, A.; Kobayashi, S. Optical characterization of liposomes by right angle light scattering and turbidity measurement. *Biochim. Biophys. Acta* **2000**, *1467*, 219-226.
